# Monthly Summary

**Published:** 1880-06

**Authors:** 


### MONTHLY SUMMARY.

*Cure of a Traumatic Salivary Fistula.*—In the *Medical Press and Circular*, February 18, 1880, we find a report of the following case by Mr. Henry Morris:—

A shoemaker came under my care in the Middlesex Hospital on June 24, 1879, for bleeding from several incised wounds, one of which was about two inches long, and vertical in direction, on the right cheek. On June 28th saliva was seen trickling from the lower end of this wound, which was all but healed. The patient now stated that since the injury the right side of the mouth felt very hot and dry, and that he had constantly to moisten it by carrying the fluid from the left side over to the right of his mouth. A fine cat-gut bougie was introduced at the oval aperture of the duct, and passed onward toward the parotid gland until it emerged on the cheek through the fistula. The newly-healed wound was laid open, and the proximal end of the injured salivary duct searched for, and found by squeezing the parotid gland until a little saliva was forced out of the duct. Into this the point of the bougie was inserted and pushed onward for about half-an-inch. The edges of the wound were brought together with three hare-lip pins and twisted sutures, and the bougie cut short, so that a short end, not long enough to be caught between the molar-teeth, was left protruding into the mouth from the slit-like orifice of the duct. The patient at once appreciated a marked difference in the feeling of the right side of the mouth, which was now in all respects like the other side, and he could distinctly feel saliva trickling into it. On July 4th the bougie came away accidentally. The wound on the cheek had been all but healed for four days, but a little discharge still escaped at its lower end. On July 9th a little saliva was escaping still: cotton wool and collodion were therefore continued over the wound until the 15th of July. By this date the wound was securely healed, and all the saliva flowed freely into the mouth. The man was retained at the hospital for a few days longer, and was then discharged well. The facility with which a bougie can be passed along the parotid duct from the mouth encouraged the author to think that the same treatment would prove successful in most cases of parotid salivary fistula, meaning by salivary fistula a fistula of the parotid duct as distinct from a fistulous communication with the parotid gland itself, such as occasionally follows abscess below or behind the ear; and also as distinct from an opening into the cavity of the mouth, such as sometimes occurs after necrosis of the jaw, and in cancer. If the orifice of the duct, which is its smallest part, becomes obstructed after long disease, the mucous membrane of the cheek may be punctured, and then a bougie might be passed into the dilated part of the duct, which is just where it pierces the buccinator muscle, and urged along it beyond the fistulous opening. In operations for the removal of tumors of the face, when an incision must be made so as to divide the duct, it would be well to pass a parotid bougie while the patient is still under chloroform, so that the potency of the duct and the flow of saliva into the mouth might be secured during and after the healing of the wound —*Medical & Surgical Reporter.*

---

*The Use and Abuse of Tobacco.*—From the earliest ages narcotics have been used in various forms by the human race, and to this practice amongst the heathen, Isaiah probably alludes when he upbraids the Jews for "remaining in the groves and lodging in the monuments;" for at that time it is well known that men went into the heathen temples, to place themselves under the influence of some narcotic, presumably opium; and so with other races. In the South of Asia the betel leaf, with areca nut and a little "chunnum" (lime produced from burning shells,) is said to act on the nervous system by soothing, and at the same time promoting digestion, the latter action is open to doubt. The cocoa leaf has likewise been much vaunted, but in this country has not proved so valuable as it is said to be in Peru, where the miners use it regularly, chewing it with lime. Tobacco is one, and one of the least harmful, of those agents by which soldiers and others who are compelled to undergo strenuous exertions at times, with a sadly insufficient supply of food, are enabled to hold out and do their work effectively. What opium is to the Tartar courier, or cocoa leaf to the Peruvian miner, tobacco is to the British soldier and sailor, in supporting him under severe and continuous efforts, when rest and sufficient food are alike beyond his reach. But the pernicious habit of smoking at any and at every time, as is practiced in the present day by persons in full health, is a useless, dirty, and an expensive one, and is too often injurious, in an indirect manner, by its acting as an inducement to drink. It is true that the more the wants of man are multiplied, the more industrious he becomes; but the use of tobacco is at once a dirty and an offensive luxury, and, with the exception of ardent spirits, there is hardly any article in which the money of the poor would not be better expended. There can, I think, be no doubt that the habit of smoking is spread more from the force of example than from any beneficial results produced by it. As regards the physiological action of tobacco upon the bulk of mankind, and apart from its moral influences, it may be received as characteristics of this substance amongst narcotics, 1st.—That its greater and first effects are to assuage, and allay, and soothe the system in general with a temporary annihilation of thought. 2nd.—That its lesser and second or after effects are to excite and invigorate, and at the same time to give steadiness and fixity to the powers of thought. The most familiar of the physiological effects of tobacco are those experienced by young smokers, who rarely fail to poison themselves to a greater or less extent, nausea, giddiness, cold sweating, and vomiting being the symptoms in their first trials. According to a celebrated writer, speaking on the effects of smoking, he states that he has repeatedly asked the Turks what they had been thinking about whilst smoking? The answer was: "Of nothing." Not a single idea could be recalled to their minds. This may be a peculiarity of the Turkish or Moslem character, but I have heard fellow-students remark that they could do no real study while smoking. However, it is a known fact that some German writers invariably smoke while writing. If it is so conducive to thought, why should it be so exclusively enjoyed by the male sex? I will conclude with an extract from the "Counter-blast to Tobacco"—"Have you not reason, then, to be ashamed and to forbear this filthy novelty, so basely grounded, so foolishly received, and so grossly mistaken in the right use thereof? A custom loathsome to the eye, hateful to the nose, harmful to the brain, dangerous to the lungs, and in black stinking fume thereof nearest resembling the horrible Stygian smoke of the pit which is bottomless."—*W. R. Glover in Druggists' Circular.*

---

*The Value of Revaccination.*—The *Medical Times & Gazette* informs us that while smallpox is prevailing extensively amid the civil population of Paris, so as to give rise to seventy or eighty deaths weekly, it is almost absent in the large military force which constitutes the garrison. This is entirely due to the rigidity with which revaccination is enforced. It is stated that there are not more than twenty cases in the military hospitals, most of them being only examples of slight varioloid, and no deaths having occurred. Every year for some time past revaccination is enforced on the arrival of the contingents; and a great number of men have been revaccinated this year in whom revaccination did not succeed last year. Every revaccination is performed from children's arms, whose parents are paid fifteen francs per sitting. What few cases of smallpox do now occur take place among the officers, or in obstinate or timid men who manage to evade revaccination.—*Medical and Surgical Reporter.*

